# Perspectives on barriers to traditional sources of sexual and reproductive health information and services: Are mHealth technologies the answer?

**DOI:** 10.34172/hpp.42607

**Published:** 2024-10-31

**Authors:** Alexander S Laar, Melissa L Harris, Clare Thomson, Deborah Loxton

**Affiliations:** The University of Newcastle, Australia, School of Public Health and Medicine, Centre for Women’s Health Research, Faculty of Health and Medicine, Hunter Medical Research Institute, Callaghan, New South Wales 2308, Australia

**Keywords:** Barriers, Challenges, Healthcare providers, Information sources, Rural Ghana, Sexual and reproductive health, Young people

## Abstract

**Background:**

In Ghana, several qualitative studies have explored users’ perspectives on conventional sources of sexual and reproductive health (SRH) information and factors which influence provision of and access in rural settings. However, there is a dearth of qualitative studies on healthcare provider (HCP) perspectives on factors that deter access to conventional sources of SRH information among young people in rural Ghana and innovative ways for addressing barriers. This study explored perspectives on barriers to traditional sources of SRH information and services and innovative ways of using mHealth technologies for addressing provision and access challenges among young people in rural Ghana.

**Methods:**

This study used a qualitative approach using in-depth interviews. Semi-structured in-depth interviews were conducted with HCPs in rural areas in three regions of Ghana between May and August 2021. Participants were selected from rural communities using the convenience snowball sampling and were interviewed via Zoom. The interviews explored the experiences and perceptions of HCPs on conventional SRH information and services and young people’s access to this information and services. The interviews were audio recorded and transcribed verbatim. Data were analysed thematically using NVivo software version 12, following the approach outlined by Braun and Clarke.

**Results:**

Twenty HCPs were interviewed for this study. The participants identified different sources of SRH information and services used by rural young people. Peers or friends, health facilities, health providers, and community settings were reported as the main services and sources of SRH information. Participants reported several barriers and challenges to the provision of and access to SRH information to young people, including socio-cultural norms, religious beliefs, unfriendly health facility environments, negative health providers’ attitudes, lack of privacy and confidentiality resulting in unfriendly youth SRH services, distance, and financial challenges due to costs of transportation which limits rural young people’s access to, and use of, SRH services. All the participants indicated that in addressing provision and access barriers, the use of mobile phones could be beneficial.

**Conclusion:**

This study highlights several barriers and challenges that deter provision of, and access to, SRH information and services for young people in rural Ghana. The findings indicate the use of innovative mobile health (mHealth) technologies may be one solution to some of the barriers and challenges.

## Introduction

 In many low- and middle-income countries (LMICs), such as Ghana, sexual and reproductive health (SRH) needs of young people (those aged between 10 and 24 years)^1^ are unmet.^2^Young people aged between 10 and 24 years^1^ who reside in LMICs constitute over one quarter of the population.^3^Young people who reside in rural settings in LMICs, including Ghana, have low uptake of SRH information and services, despite the benefits they can provide.^4-11^ In Ghana, despite successive government efforts to ensure universal access to SRH information and services (a core outcome of the Sustainable Development Goals (SDGs), barriers to conventional SRH services still remain.^12^ The under-utilization of SRH services has been explained, in part, by multiple access barriers including social and religious norms and beliefs,^13-15^ unfriendly attitudes of health providers,^13-15^ limited health infrastructure, and long distances to healthcare facilities and associated travel costs.^13-16^

 Despite the implementation of Health Service Policy and Strategy for youth-friendly services over the years to ensure that young people have knowledge of available SRH services and information,^17^ young people, especially in rural settings of Ghana, often encounter multiple barriers and challenges to the access and uptake of SRH information and services.^13,18,19^ In rural Ghana, gaps still exist in coverage, universal access, and youth-friendly SRH services. In Ghana, current conventional SRH interventions have not adequately addressed young people’s SRH information needs in rural settings, resulting in a clear unmet need.^20-22^ The high unmet need for SRH information and services among rural young people in Ghana results in high rates of unsafe abortion, early and unintended pregnancies, unplanned births, sexually transmitted infections (STIs), including human immunodeficiency virus (HIV), and increased morbidity and mortality.^23^ This is partly explained by multiple barriers young people encounter in accessing SRH information and services, including lack of awareness of where to access information and services, inadequate physical space resulting in fear of lack of privacy and confidentiality,^13^ distance to health facilities and cost of services,^13^ negative health providers’ attitudes,^13,15^ community members’ negative perceptions and stigma^13,15^ socio-cultural norms and practices,^13,18^ religious beliefs and practices,^18^ and government policies,^24^ resulting in poor access to SRH health information and service utilisation among rural young people.^13,15,18,25^

 In Ghana, the contraceptive prevalence rate among all women is 25%, with only 20% using modern methods.^26^ The majority of women continue to use less reliable traditional methods, such as rhythm, withdrawal, and folk methods.^26^ Unmet need for effective contraception is higher in rural areas for both married and unmarried young women (45.7%) in Ghana.^27^Despite an increase in the proportion of sexually active women and men in Ghana,^28^ evidence suggests that the use of modern contraceptive methods among women aged 15-49 is low, especially in rural areas,^12,28^ largely due to inadequate knowledge.^29^

 One of the key targets of Ghana’s Adolescent Health Service Policy and Strategy is to ensure that 90% of adolescents and young people have adequate knowledge of SRH services and information.^17^ Achieving this target requires addressing the myriad inequities to the access and use of SRH information and services through innovative approaches. Use of innovative mobile health (mHealth) technologies present critical opportunities for the achievement of the SDGs and universal health coverage (UHC) agendas for SRH services for rural populations in LMICs. Whiles there is a growing body of qualitative evidence in rural LMICs regarding barriers and challenges to conventional SRH information and services, identifying the potential and important role of innovative mobile phone technologies in addressing these challenges among rural young people,^30,31^ this information is yet to be explored in rural Ghana.

 In rural Ghana, several qualitative studies have explored the perspectives of health service users and young people, and the barriers to, and challenges of, conventional sources of SRH information and services are well documented.^13,15,18,25^ However, there is a dearth of qualitative studies on healthcare provider (HCP) perspectives on factors that deter access to, and use of, SRH information from traditional sources among young people in rural Ghana and how health providers are using innovative approaches for addressing barriers. This study obtained information on HCP perspectives regarding access barriers to the delivery of SRH information and services to young people through traditional face-to-face sources and whether innovative mHealth technologies are a solution for addressing these barriers in rural Ghana. The perspectives of HCPs delivering SRH information and services to young people in rural Ghana via mobile phone platforms were explored qualitatively. The implementation of a qualitative approach for this study facilitated the flow of communication and interaction between the researcher and the participants, resulting in the sharing of rich, detailed information regarding their views and experiences through the use of an open-ended semi-structured interview guide.^32,33^

## Methods

###  Study design

 This study used a qualitative design with an in-depth semi-structured interview guide to address the research questions and to comprehensively explore HCPs’ views on the phenomenon under study. A qualitative research approach was chosen as it provides participants with the opportunity to express themselves and share their varied range of experiences and viewpoints in a detailed and in-depth manner.^34^ The interview guide was developed in English, being the official language of Ghana). The interview guide was pre-tested with a similar population to that from which the sample was drawn in a similar environment to help strengthen the tool.

###  Study setting

 The study was conducted with HCPs located in three rural regions of Ghana (Volta, Upper East, and Northern Regions). These regions were purposively selected based on Savana Signatures SHE + mHealth SRH intervention sites for youth population. These rural regions have limited healthcare infrastructure and health personnel, and the distance to healthcare facilities is far above the World Health Organization recommended distance of five kilometres,^16^ thus creating inequalities in access to health services and information.^35^

###  Data collection

####  Study participants recruitment process

 Qualitativein-depth interviews were held with HCPs between May and August 2021. The participants were first approached by the management of Savana Signatures, a community non-governmental organisation implementing mHealth Sexual Health Education Plus (SHE + ) health promotion education for young people in rural communities in Ghana. Interview materials including flyers, a letter of invitation, and an information statement were emailed to the potential participants by the site coordinator of Savana Signatures. The letter of invitation informed potential participants about the purpose of the study, recruitment procedures, and the researchers involved, as well as study requirements, confidentiality provisions, and potential risks associated with participating in the study. Contact details of the PhD candidate and principal supervisor were provided for participant queries.

 Participants were recruited using a convenience snowballing method,^36^ where current research participants assisted in the recruitment of new participants from among their colleagues.^37^ Participants who agreed to take part in the study were sent a Zoom link via email. At the beginning of the Zoom conversation, participants were given a further opportunity to ask any questions before providing informed consent. For HCPs wishing to participate in the study, a time convenient for the participant was scheduled. At this time, participants were reminded to let their colleagues know about the study. Recruitment continued until the point of data saturation.^38,39^ Data saturation is where adding more participants to the study no longer results in additional perspectives or information.^38,39^ The sample size of 20 participants, comprising13 females and 7 males, was large enough to sufficiently describe the phenomenon of interest of this study, to address the research questions, and to reach the point of data saturation, as required in qualitative research.^38^ This helped prevent the collection of repetitive data that did not add any value to the research. The total sample for the study was deemed to be adequate for sampling among a relatively homogenous population of HCPs and achieved thematic saturation.^40,41^

###  Data management and analysis

 A thematic analysis approach was employed following rigorous qualitative induction data analysis stages as outlined by Braun and Clarke.^42,43^ All the recorded audio interview data were first listened to and transcribed verbatim. Prior to the analysis, the transcripts were read and re-read several times to ensure familiarization with the entire data to ensure that every aspect of the information recorded is captured.^44^ Notes of initial ideas were made in a memo created in an NVivo journal to define the unit of analysis.^42,44^ The data were imported into the qualitative management program NVivo v.12 (QSR International Pty Ltd) for coding and analysis.^45^ The transcripts were coded by generating labels to attach to text segments that emerged as an important user viewpoint in the transcripts and categorised these in relation to the concepts.^42-44^ Codes were generated by delving deeper into the data by reading the transcripts line by line and assigning codes to paragraphs or segments, moving back and forth in between the data as concepts unfolded and meaning emerged from the dataset.^42,46^ Reflection on the participants’ own words, phrases, and experiences was followed by the categorisation of codes into themes and sub-themes relevant to the research question in an inductive manner.^42,46^ The themes were further reviewed to identify meanings and relationships between themes. This process ensured that the themes accurately reflected the perceptions and experiences of HCPs on the phenomenon. Extracts or quotes from the transcripts that best explained each theme and sub-theme for the interpretation of the data were used in the write-up. The authors used a unique pseudonym for interviewees to protect their identity.

## Results

###  Participant demographic information

 A total of 20 HCPs were interviewed. Thirteen of the participants were female and seven were male, with ages ranging between 25 and 42 years. The participating HCPs included medical doctors (3), nurses (9), midwives (2), community/public health nurses (5) and allied HCPs (1), with health care delivery experience spanning between two and 16 years. For the 20 participants two were from Volta region and 9 each from Northern and Upper East regions. The majority (17) of the participants were Christians.

###  Study themes

 In this section, information on HCPs’ knowledge regarding conventional sources of SRH information available to young people in rural Ghana, and barriers to the provision of, and access to, SRH information and services was captured. The results indicated a wide range of SRH information sources used by young people, and a similarly wide range of conventional service types and the barriers to these, including overarching themes that centred on health providers’ attitudes, health facility environment, geographical distance, and cost and community factors. These themes are reported in detail, with illustrative quotes from participants, in the following sections.

####  Sources of SRH information for young people

 In this study, the participants reported the most used sources of information for SRH information and services by young people in rural Ghana were health facilities, health providers, community members, and peers or friends. However, other sources were also mentioned, such as community radio stations, community durbars, schools, non-governmental organizations (NGOs), television, and traditional birth attendants (TBAs). Using these sources, young people sought SRH information and services on contraception, family planning, and HIV and other STIs. The key and most used sources for SRH information by young people, together with their motivations for using these sources, are reported with illustrative quotes below.

####  Friends and peers

 Participants reported that young people mostly sought SRH information from friends or peers in their communities due to comfort levels and the stigma associated with discussion of sexual health matters or issues in the local communities. However, the legitimacy of this information was questioned.

*“…So, if their friends have had the chance to experience a sexual health issue or information or experience concerning a certain issue, they share it with their friends but as to whether the information is authentic or not is a different thing altogether…”* (HCP E, Midwifery Officer).

####  Community settings 

 Within the community settings, family members, radio stations, community durbars, schools, NGOs, and TBAs served as sources of SRH information for young people. However, participants also reported that these sources were not frequently used due to social stigma.

*“…I think ehh, some get their sexual and reproductive health information from the communities...from family members, radio stations, community durbars…Others also get it from some specialized organizations that are interested in sexual and reproductive health matters, such as non-governmental organizations...”* (HCP H, Clinical Health Psychologist).

*“...some youth get their information from local midwives called TBAs and herbalists in their communities…”* (Participant A, Community Health Nurse).

 Some participants indicated that within the family, typically the mothers or fathers transfer information on SRH issues in line with societal norms.

*“...in the communities, we have the parents and other community members [provide] education [to] young people related to sexual and reproductive issues as they develop into puberty...to help them integrate well into the societal norms as prescribed and transferred from the family to the young girls and boys”* (HCP F, Gynaecologist).

####  Health facilities 

 Participants reported the use of community health facilities, such as health centres and clinics, where young people obtained SRH information and services in person from HCPs.

*“…some of them access it from the community health facilities such health centres and clinics...” *(Participant B, Health Counsellor).

*“…some of them access it from the health care providers in the health facilities and during outreach at community durbars...” *(Participant B, Health Counsellor).

####  Barriers and challenges to provision of, and access to, SRH information and services

 Aside from sources of information, HCP knowledge regarding the barriers young people encounter in accessing SRH information and services from rural Ghana was also explored. All participants reported several factors which influenced provision of, and access to, SRH information and services among young people. Below are some general excerpts on barriers and challenges from a health counsellor.

*“…in our communities, there are multiple barriers and challenges including cultural and religious issues…cultural and religious issues do not make it easy for we the health providers to give counselling information or education on sexual and reproductive health services to young people in the community…”* (Participant B, Health Counsellor).

*“…aside cultural and religious barriers and challenges…inaccessible communities and lack of health facilities due to long distance, poor roads and cost make these services not accessible and available to young people in our communities …”* (Participant B, Health Counsellor).

 These general factors on barriers and challenges above are also detailed below.

####  Community barriers

 Community factors centred on socio-cultural norms and religious beliefs. Participants reported that, in rural Ghana, the discussion of sex and related issues among young people is often frowned upon by both traditional norms and religious beliefs. Traditional social norms largely perceived the discussion of sex-related issues as only suitable for married people. Religious beliefs were reported to discourage young people from using contraceptives. Participants cited the Catholic religion as a classic example that discourages use of contraceptives and family planning services. Participants indicated these factors served as important barriers that prevent the provision, access, and use of SRH information and services among young people in their communities.

*“Yeah, the challenges are, I think if you look at the cultural perspective, the Ghanaian society for instance, sex education is not something that many people want to talk about or educate young people on…sex education raises cultural issues and [is] frown[ ed ] upon by community members...”* (HCP H, Clinical Health Psychologist).

*“...because of the religious issues…it is not possible to go into a community and start talking about sex and condoms…those things [are] seen as taboo in the communities…the social and religious beliefs on these issues do not permit sexual health education with young people...Community members regard young people as being bad or immoral”* (HCP D, Health Counsellor).

####  Geographical distance and cost barriers

 Participants reported long geographical distance and cost to health facilities as a major challenge for seeking SRH information and services from health facilities by young people in rural Ghana. Participants indicated that the few health facilities available in rural communities are sited in the district capital and towns far away from the remote communities. Therefore, to seek SRH care requires some travel and cost for transportation and services. This puts financial pressure on vulnerable young people when seeking information from other health and non-health related sources.

*“...some of the SRH services are not available at the communities. At the places where they [are] available [it] require[s] travel and transportation cost[s]…the cost compels some young people [to] not visit health facilities for formation on SRH…”* (Participant C, Call Centre Counsellor).

*“…so those who cannot afford the cost for services decide to stay away from visiting the health facility or explore alternative sources available to them such as TBAs or local midwives or peers in the communities”* (Participant A, Community Health Nurse).

####  Attitudes and characteristics of health providers that deter or prevent SRH service use

 Participants reported negative attitudes or behaviours of HCPs as a barrier to provision and use of SRH information and services by young people. Participants related negative behaviour to HCPs’ judgements of young people visiting the health facility for information on their sexual health as being immoral or promiscuous. They stated that in certain instances some health providers neglected to offer certain services. Some health providers refuse to pay the necessary attention to the young people attending the service, making them to wait longer for services than expected, as a way of punishment or discouraging them from visiting the health facility for sexual health information and services. In this study, participants also stressed that the judgemental attitudes of HCPs served as deterrents to seeking SRH information and services from health facilities by young people in the rural communities.

*“…Some of the health providers are not welcoming at all…they stigmatise young people who visit the health facility for sexual and reproductive services as been spoiled or promiscuous…some even refuse to provide them with the information they need.”* (HCP F, Gynaecologist).

*“…the way health providers handle the young people stigmatise [s], discourage[s] them, especially the timid or shy ones from visiting the health facilities to seek information for sexual health services…”* (Participant L, Medical Officer).

 In addition to negative attitudes of health service providers, participants reported the gender or sex of the service provider as a factor influencing the seeking of SRH services by young people.

*“Some young people feel shy or uncomfortable consulting health providers of the opposite sex…they prefer [a] same sex provider”* (Participant B, Health Counsellor).

 Also, related to health provider factors, participants also reported that the age of HCPs influence SRH information and service-seeking by young people. Participants stated that some young people felt older providers did not show compassion when they were approached for SRH care.

*“…young people are not comfortable seeking or discussing their SRH issues with health providers who are too old or aged due to their unfriendly posture…young people regard some of them as being difficult or not having compassion for them…”* (Participant C, Call Centre Counsellor)

####  Health facility barriers

 Participants reported that an unfriendly health facility environment hampered provision of, and access to, SRH information and services for young people. They raised concerns about a lack of privacy and confidentiality and stated that young people were not comfortable seeking services in a setting crowded with clients who were their fellow community members, due to stigma and confidentiality issues. This is demonstrated in the quote below.

*“The structures of the health facilities in our settings are not conducive to the provision and use of sexual health services for young people…there is no privacy due to [a] lack of youth corners…they are not comfortable mixing with the other clients.” *(Participant G, Public Health Nurse).

 With regard to unfriendly health facility environments, a community health nurse remarked that it is those young people who are brave and not timid who visit the health facilities for SRH services.

*“…because of the stigma society [has] attached to sexual health issues…the young people who are bold enough go to the health facilities to seek for information…”* (Participant A, Community Health Nurse).

 However, regarding these sources, some participants were of the view that some young people are uninformed about where to obtain sexual information in their communities.

*“…I don’t think some of them have any knowledge on a source where to get sexual health information in the community”* (Participant P, Midwife).

 In this study, all the participants reported that some young people contact health providers on their SRH issues using a mobile phone.

*“…some of the young people now contact some of the health providers using the mobile phone for information and services on a range of their sexual and reproductive health issues across our communities…”* (Participant A, Community Health Nurse).

 All the participants also acknowledged that using mobile phones help address some of the conventional challenges of access to, and provision of sexual and reproductive health information and services among young people in their rural communities compared to the in-person services. Below is an excerpt from by a counsellor.

*“…using the mobile phone among providers and young people alongside the in-person services help address some of the conventional challenges…”* (Participant C, Call Center Counsellor).

## Discussion

 This study presented findings on HCPs’ perspectives on the conventional sources of SRH information and services for young people and their limitations in rural Ghana. Participants identified that the most used sources of SRH information were health facilities, health providers, community outreach and durbars, as well as peers or friends. Additional SRH information sources cited included radio stations, schools, NGOs, television, TBAs, and communication technology. Similar studies have reported health facilities, health providers, community settings, and friends as common sources of SRH information and services for young people in rural Ghana.^13,15,18^ A scoping review has identified these factors as challenges contributing to low rates of access to SRH information and services among young people in rural LMICs.^47^

 Participants also reported barriers to formal SRH services, which included community factors centred on social taboos and religious influence, inaccessible health facilities due to distance and cost, gender of health providers, HCP attitudes, and limited youth-friendly services or spaces at health facilities resulting in a lack of privacy and confidentiality. Similarly, a qualitative study which explored perceived barriers to accessing and using young people’s health services in rural Ghana, found community and health facilities, peer influence, judgemental attitudes of providers, and inadequate physical space and privacy barriers restricted access to, or use of, health services among young people.^13^ The findings are also consistent with a qualitative study which explored health care provider perspectives on barriers to, and enablers of, family planning use in rural Pakistan and found a lack of adolescent-friendly spaces for counselling for reproductive health services, as well as discriminatory gender norms and cultural practices as barriers to the uptake of modern contraceptive services.^48^ Ascertaining barriers and facilitators for accessing SRH information and services is important to ensure equitable access by young people in rural settings in LMICs.^30,49,50^

 A key finding of the study is the use of peers as a preferred source of SRH information by young people. This finding is consistent with those of a qualitative study from rural Ghana, which explored young people’s reproductive health knowledge, choices, and the factors that affect these choices, found that a majority of young people relied on their peers for SRH information.^51^ Similar findings have been reported among young people in other rural LMICs.^52,53^ Research from LMICs has demonstrated that young people seek SRH information from their friends and peers due to the comfort associated with discussing shared experiences related to their sexual health, compared to the stigma associated with obtaining this information from health providers and community members.^54,55^ The current study also found that some rural young people are unaware of where to access SRH information in the community, despite the need for these services. This finding is consistent with a qualitative systematic review which explored factors influencing access to, and use of, SRH information and services among young people in LMICs, who reported the lack of knowledge of available SRH services as a barrier.^56^ Despite the global agreement regarding access to, and use of, SRH information and services as a basic human right of young people, universal access and use of these services remains low in rural settings, including those in Ghana.^18^

 Although numerous sources of SRH information and services are available for young people in rural Ghana, this study found several community, health care provider, and personal barriers and challenges which influenced its provision and access among young people. Some of these factors included community barriers, such as religious beliefs and social norms, and distance and associated transport costs to health facilities, as well as health facility barriers, such as negative attitudes of health providers and unfriendly health facility environments. Several studies in Ghana have reported similar findings regarding the barriers and challenges to the provision and access of SRH information and services among young people in rural Ghana.^13,16^ In particular, another Ghanaian qualitative study,^13^ found unfriendly health facility environments, long patient waiting times for services, and inconvenient health facility opening hours as barriers affecting access and use of SRH services. Similarly, a qualitative systematic review, which synthesized the barriers to accessing SRH services among young people in LMICs, identified an unsupportive health facility environment as a key barrier.^57^ In rural LMICs, such as Ghana, young people ascribe much importance to their SRH privacy and may only access services if they perceive that their privacy will be guaranteed. Addressing privacy and stigma issues at the health facility level requires the establishment of suitable youth-friendly spaces.^13,15^

 The unwelcoming attitudes of HCPs were also identified as a major barrier to young people’s use of SRH information and services at health facilities. Some HCPs attributed their negative manner to their judgemental and reprimanding attitudes towards young people for visiting the health facility for SRH information and services. Some health care providers described young people who visited the health facility for contraceptives and family planning advice as immoral or promiscuous. This finding aligns with a similar study in rural Ghana which assessed the opinions of service providers on delivering SRH services to young people and found the negative attitudes of service providers resulting in poor utilization of SRH services by young people.^15^ Further, a recent narrative review involving LMICs also reported provider prejudice as a factor which compromised young people’s access to, and use of, SRH services.^58^ Several other qualitative studies in rural LMICs have also reported negative health providers’ attitudes as a barrier to young people seeking SRH services.^59-62^

 In this study, perceived community stigma emanating from negative attitudes of community members acted as a major barrier to the use of SRH information and services by young people. These finding are consistent with other qualitative studies in rural Ghana, which have found the discussion of issues relating to sex and related sexual health issues, such as contraceptives and family services, was perceived as only appropriate for married people.^13,19,51,63,64^ Similarly, other studies have also reported negative perceptions of community members as being a barrier to the use of SRH services in rural LMICs.^60,65^ In the rural Democratic Republic of Congo, a similar study, which used focus group discussions among young women, found sociocultural norms, male dominance in family planning decision-making, and pressure from family members influenced the use or non-use of modern contraception.^66^ Guaranteeing access to youth-friendly and acceptable SRH services among rural young people is vital for promoting equitable access and effective use of SRH information and services.^49^ Addressing community issues relating to social and religious norms and beliefs is therefore key to creating a supportive environment, tailored to young people’s needs for SRH services and information in rural settings.^15,65,67^

 Despite the range of sources reported by participants, this study found some HCPs and users are using the mHealth technology for SRH services, since it was more user friendly. However, procedures need to be taken for the expansion of mHealth resources and their integration in the health system.^30,68^ A population-based study, which explored the perspective of healthcare users and providers in rural Ghana, found mHealth technology was a viable tool for lessening conventional barriers relating to access to, and provision of, SRH services.^69^ The use of mobile phones for this purpose is found to be feasible and effective in addressing conventional barriers and challenges to SRH services in rural LMICs,^30,70^ by providing a platform for equitable access to such services by vulnerable young people.^18,19,71^Expanding access to SRH information and services through mHealth technologies is vital to achieving one of the SDG targets, to establish UHC for SRH information and services.^72,73^

## Strengths and limitations

 This study has some limitations. The key limitation of this study was the inability to include young people’s perspectives on SRH services, due to the COVID-19 pandemic, which meant no young people could participate in the interviews. The findings were just a conjecture from HCPs perspectives. Also, the HCPs were not a representative sample of all HCPs providing mHealth-based SRH services in the rural communities in these regions and others may have different perspectives. This may have implications in understanding young people’s experiences on the use of mHealth technology for SRH information and services, and their perspectives on improving access to SRH services in rural Ghana. Future research should therefore include young people. Obtaining young people’s views could have provided important additional information. Also, the use of Zoom video-calls, rather than in-person interviews, could have led to the exclusion of potential participants who did not have access to Zoom facilities. These limitations notwithstanding, our study findings provide useful insight into current sources of SRH information and services for young people in rural Ghana, and barriers to accessing to conventional services, from the perspective of HCPs, who are the gatekeepers of SRH care. The study findings provide crucial insight for the government, stakeholders, and policy makers in Ghana for the need for establishing innovative mHealth initiatives to achieve universal access to and utilization of SRH services among young people in the rural Ghana.

## Conclusion

 This study highlights several barriers and challenges that affect the provision of, and access to, SRH information and services for young people in rural Ghana, and also identifies the need for innovative initiatives. Given the local nature and importance of these issues, innovative mHealth technology solutions could address some of the perceived barriers to access to, and use of, SRH information and services among young people. These findings can provide information for policy makers, HCPs, and other relevant stakeholders about the need for the implementation of innovative policies to address conventional barriers to enable young people’s access SRH information and services in rural Ghana. These findings also have the potential to guide policy makers and implementers in adopting innovative mHealth initiatives for health system strengthening for addressing conventional barriers to facilitate access to, and use of, SRH information and services among young people in rural contexts of Ghana.

## Acknowledgements

 This research was supported by an Australian Government Research Training Program (RTP) Scholarship for my PhD program. We would like to acknowledge Australian Government Research Training Program support for my PhD studies and the staff of Savanna Signatures of Tamale and the research participants for their supportive role during the data collection for my dissertation.

## Competing Interests

 None declared.

## Ethical Approval

 Approval for this research was obtained from the Human Research Ethics Committee of the University of Newcastle (H-2020-0249) and the Institutional Review Board of the Navrongo Health Research Centre of the Ghana Health Service (NHRCIRB406). Verbal informed consent was obtained from all participants in the English language. The informed consent emphasised the voluntary nature of participation, the recording and transcription and assurance with respect to anonymity and confidentiality of their data. All participants provided oral consent, and their rights of confidentiality and anonymity were emphasised. All interviews were conducted in accordance with the University of Newcastle’s Human Research Ethics Committee Policies. Electronic data of the study was stored on password protected computers, accessible only to the study team.
